# Examining speakers’ subjective and bio-behavioral responses to audience-induced social-evaluative threat via immersive VR

**DOI:** 10.1038/s41598-026-38915-8

**Published:** 2026-02-06

**Authors:** Sue Lim, Ralf Schmälzle, Gary Bente

**Affiliations:** 1 The Brian Lamb School of Communication, Purdue University, 100 N. University St., West Lafayette, IN 47907 USA; 2 Department of Communication, Michigan State University, East Lansing, MI 48824 USA

**Keywords:** Virtual reality (VR), Social-evaluative threat, Public speaking, Communication apprehension, Science communication, Social cognition, Neuroscience, Psychology, Psychology

## Abstract

Success in public speaking hinges on engaging an audience, which involves a high-stakes social interaction process that remains a significant source of anxiety and stress for many. Using a virtual-reality (VR)-based experimental paradigm, we tested how speakers delivering scientific talks perceive and respond to supportive vs. unsupportive audiences. We collected behavioral (gaze, paralinguistics, motion expressiveness/openness), physiological (heart rate, electroencephalography, breathing rate, pupil dilation), and self-report measures to assess audience effects. The unsupportive audience elicited greater negative affect, arousal, and anxiety, and higher perceived cognitive and social effort. Physiologically and behaviorally, speaking to the unsupportive audience decreased the speaking rate. Acoustic analyses further indicated greater emotional arousal and vocal dominance in the unsupportive condition. These findings highlight VR combined with physiological measurement as a powerful approach for investigating audience effects and social-communication processes, with clear implications for augmenting social intelligence and communication skills.

## Introduction

Whether delivering a scientific talk, a business pitch, or a university lecture, public speaking is a key competency that 21st century knowledge workers need to master to succeed, but also one of the most highly ranked sources of anxiety and apprehension. Public speaking is a hotbed of social communication processes^1,2^, governed by a dynamic feedback loop where a speaker’s efforts to engage an audience are met with audience reactions that can powerfully shape their performance and trigger profound anxiety. Although the term public *speaking* emphasizes verbal communication, the uniquely social nature of public speaking is perhaps even more evident in nonverbal and paralinguistic channels: Speakers, for instance, connect with their audiences through an array of non- and paraverbal behaviors, including eye gaze, body language, or prosody.

Often overlooked, however, is the fact that audiences are also communicating with the speaker. Audience feedback occurs predominantly via social-evaluative nonverbal signals – for example, unengaged audiences make no eye contact and exhibit little positive backchannel activity (e.g., head nodding)^3^. Such social feedback signals are perceived by the speakers, which can set off a cascade of negative self-attributions, feelings and symptoms of embarrassment (e.g., blushing, dry mouth), and psychological disturbances (e.g., working memory interference, speech disfluencies)^4–9^. These effects of the audience on the speaker are the topic of the current study.

This study examines the effects of the audience’s behavior on the speaker, using virtual reality as an experimental petri dish to examine social communication processes. Theoretically, our work is informed by the biopsychological (BPS) model of challenge and threat^10^, which connects the social-evaluative nature of public speaking to people’s biopsychological responses. Methodologically, we leverage immersive virtual reality (VR) as well as physiological, behavioral, and subjective measures to comprehensively assess a broad spectrum of behavioral, viscero-motor, and social-cognitive variables under realistic public speaking conditions. The results augment existing efforts in the scientific community to capture the multifaceted aspects of social processes and build better interventions targeting public speaking anxiety.

In the following, we first summarize related works and point out the research gap, which consists of causal manipulation of social factors (i.e., the audience behaviors that form the stimulus triggering social-evaluative interpretations in speakers) and the measurement of the complex dynamics underlying public speaking in the context of science communication. We then explain how the current VR-based approach offers a unique solution. Finally, we present the current study in which participants gave scientific presentations in front of two types of virtual audiences (supportive vs. unsupportive).

### Challenge and threat in public speaking processes

The behavior of an audience is a potent form of social-evaluative feedback that can act as a significant psychological stressor, comparable to other social challenges like exclusion^11^ or stress interviews^12^. Influential frameworks, such as the Biopsychological Model of Challenge and Threat, describe how individuals respond to such stressful performance situations^10^. The model posits that individuals assess their resources and demands of a situation, leading to either a challenge or a threat response. A challenge response occurs when individuals perceive they have sufficient resources to cope with demands, resulting in adaptive physiological responses, such as increased heart rate. Conversely, a threat response arises when individuals are overwhelmed and lacking in resources, triggering negative physiological reactions, such as increased blood pressure and anxiety.

Although the challenge/threat framework was originally developed in social psychology with a focus on coping and resilience under stress, it can be applied to the public speaking situation. At the core of the challenge/threat framework lies how interpretations of challenging/threatening social situations influence physiology, with a focus on cardiovascular stress responses. While we do not directly test this model, it informs and motivates our multi-modal approach. Since public speaking is a complex biopsychosocial phenomenon, studying it requires a strategy that can capture its different facets simultaneously. Established frameworks in emotion and stress research^12,13^ suggest that relying on a single measurement modality has limitations in this regard. Self-reports provide insight into a speaker’s subjective experience but are retrospective and cannot capture real-time dynamics. Behavioral measures quantify overt performance but are ambiguous regarding the speaker’s internal state. Finally, physiological measures offer an objective window into bodily arousal but cannot, on their own, distinguish between positive states like challenge and negative states like threat. Therefore, the strength of our approach lies in triangulating these three channels – subjective, behavioral, and physiological. This integration allows us to capture a holistic picture of the speaker’s response to the public speaking challenge and their coping behaviors^13,14^.

This multi-modal perspective reveals the limitations of traditional approaches to study public speaking. Foundational research, particularly in work about communication apprehension, public speaking anxiety, and social stress has largely relied on observation and self-reported evaluations^15,16^. For instance, MacIntyre and colleagues provided participants with scenarios describing audience characteristics (e.g., responsiveness, pleasantness) and had them answer a questionnaire about their imagined audiences^17,18^. Hsu had participants give impromptu speeches in front of confederate audience members and complete a questionnaire after the task^19^. However, studies that solely use self-reports do not capture the hidden neurophysiological and overt behavioral phenomena that are ongoing as the public speaking situation unfolds, such as how speakers respond to audience reactions during their live performance. Other researchers have examined speakers’ biological responses, such as cardiovascular reactivity and changes in heart rate, as they spoke in front of various human audiences^20–22^. However, these studies occurred in the lab with confederates as audiences, leading to low ecological validity and cost and time spent on the confederates.

### Current study: VR-based experimentation for examining public speaking processes

Within this context, immersive VR technology holds great potential for experimental research in public speaking. Immersive VR enables researchers to precisely manipulate experimental variables while simulating real-life environments and situations, enhancing ecological validity of lab-based studies^23^. This capability applies to social situations and cues as well. For instance, researchers can vary audience size, engagement levels, and positive and negative audience reactions and study the effects on the speaker^24–26^. When integrated with physiological and behavioral measurements such as eye-tracking and heart rate tracking, VR-based paradigms can illustrate the cause-effect mechanisms in real-time^27^. These features have led to recent research that leveraged immersive VR to study the effects of virtual audiences on speakers^7,8,28,29^.

**Fig. 1:**
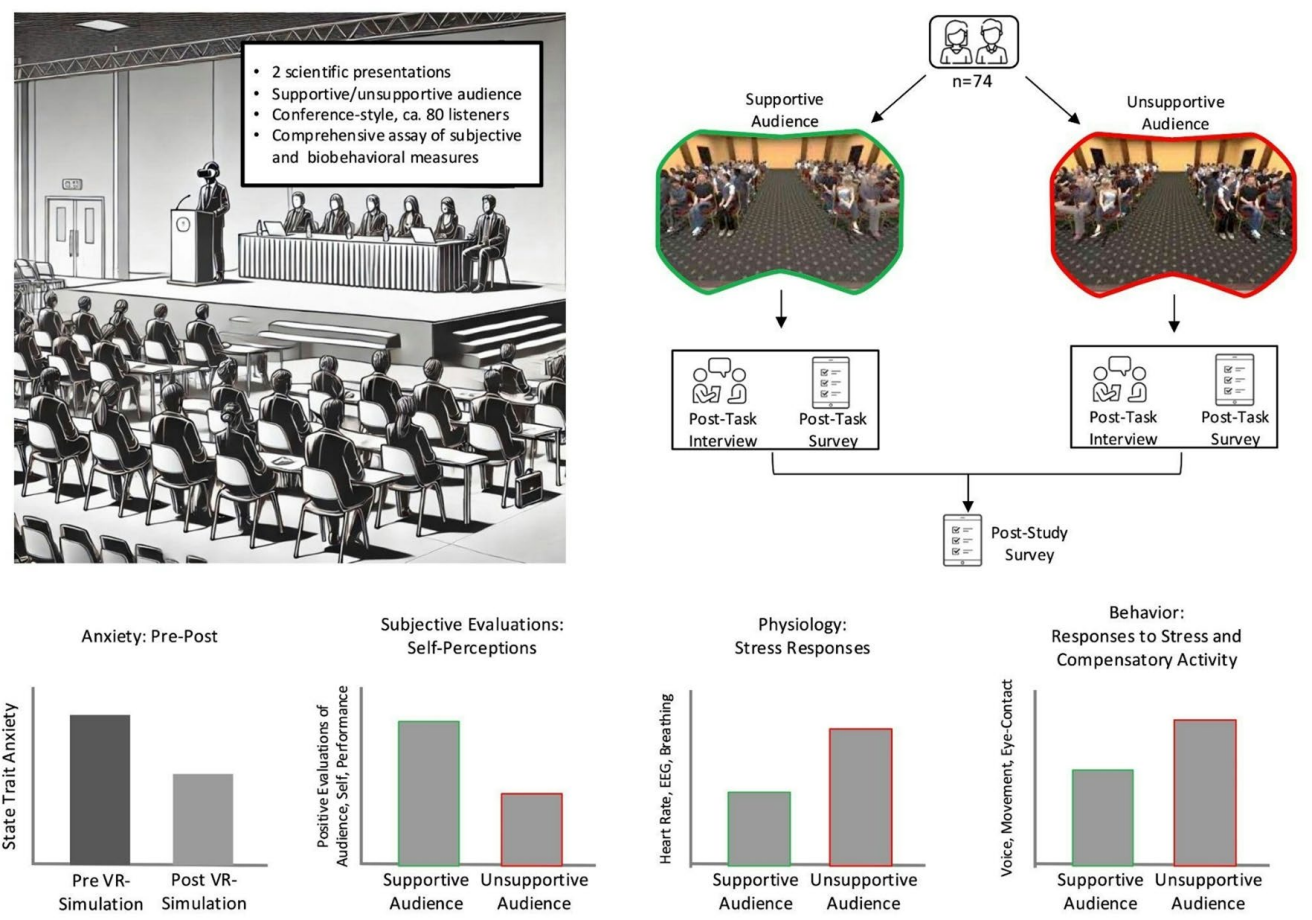
Overview of the study setup and design, main measures, and predictions. Top Left Panel: Participants were asked to come to the lab with two 8-12-minute scientific presentations prepared. During the study, participants wore the VR headset and gave two presentations in front of a large audience. **Top Right Panel**: Presentations were given to a supportive audience vs. an unsupportive audience (counterbalanced across participants). **Bottom Panel**: To comprehensively assess audience effects, we examined participants’ subjective, physiological, and behavioral responses (see the “Methods” section for details). We tested the differences between the supportive vs. unsupportive audience manipulation (bar graphs are conceptual illustrations of our predictions and do not show empirical data).

Building upon this body of literature, our study examines how speakers respond to socio-evaluative threat by manipulating virtual audience behavior in immersive VR (See Fig. 1). Specifically, participants were asked to give a research-based presentation to supportive as well as unsupportive computer-generated audiences (within-subject design) while immersed in a virtual environment that resembled a typical academic conference venue. We focus on scientific presentation because of its multifaceted nature: Effective scientific presentation requires sustained audience engagement and breaking down complex ideas for the audience in addition to interesting content itself^30^. Also, scientific presentations are often connected to important outcomes such as getting a job or persuading the audience toward a specific action, making feedback signals from the audience more salient to the speaker.

In terms of measurement, we combined multiple physiological (e.g., electroencephalography, heart rate), behavioral (e.g., expressive motion, valence in the voice), and subjective measures (e.g., self-reported anxiety symptoms experienced during the presentation), aiming to capture not only speakers’ self-reported experience after the performance, but also their neurophysiological activity and behavior during the presentations. Our main hypotheses centered around the effect of the audience manipulation on these measures. First, we made the following predictions about audience effects on people’s subjective experiences:

Hypothesis 1 (H1): Individuals will perceive greater pressure to exert (a) cognitive, (b) physical, and (c) social effort when presenting in front of the unsupportive audience compared to the supportive audience.

H2: Individuals will report greater anxiety-related symptoms, manifested through (a) language and behavior, (b) thoughts, and (c) physiology when presenting in front of the unsupportive audience compared to the supportive audience.

H3: Individuals will report having experienced (a) greater negative affect, (b) heightened arousal, and (c) lower sense of dominance when presenting in front of the unsupportive audience compared to the supportive audience.

We also expected that audience behavior will have similar effects on the following corresponding physiological and behavioral measures:

H4: The unsupportive audience will elicit (a) greater negative affect, (b) heightened arousal, and (c) lower sense of dominance in the voice when presenting in front of the unsupportive audience compared to the supportive audience.

H5: The unsupportive audience will induce elevated levels of (a) breathing rate, (b) heart rate, and (c) pupil dilation compared to the supportive audience.

Furthermore, we posed two research questions about audience effects on neural activity and speaking and nonverbal behaviors because those measures often represent a combination of complex processes:

Research Question 1 (RQ1): Will audience behavior influence individuals’ neural activity?

RQ2: Will audience behavior influence individuals’ (a) speaking rate, (b) openness in motion, (c) expressiveness in motion, (d) eye contact with the audience, and (e) gaze dispersion while presenting?

In addition to audience effects, we examined the effectiveness of the VR-based public speaking intervention in general:

RQ3: Does completing the VR-based public speaking tasks decrease anxiety levels?

RQ4: After individuals complete the VR-based public speaking tasks, how likely are they to recommend the intervention to others?

## Results

**Fig. 2:**
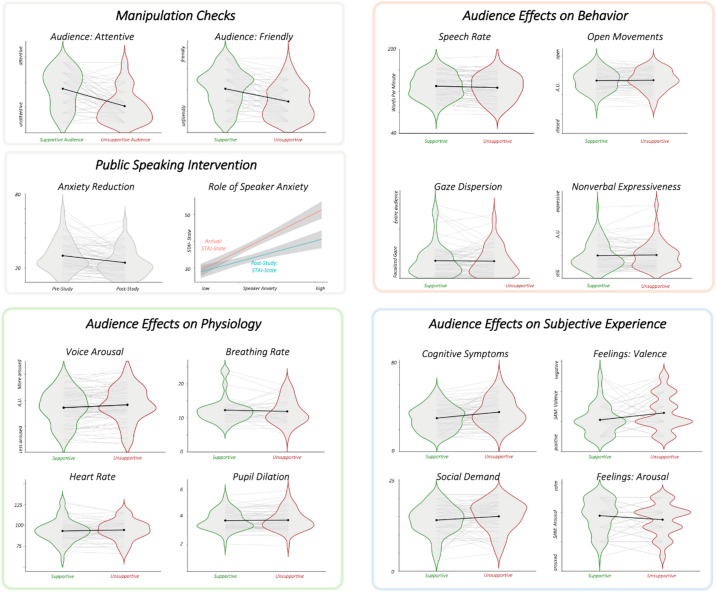
llustration of results: audience effects on speakers’ behavior, physiology, and subjective feelings. Post-study survey data confirmed that speakers were perceiving expected differences between the supportive and unsupportive audiences, in line with the intended manipulations (Manipulation Checks Panel). Results also revealed reductions in anxiety levels, and stronger reductions on the basis of public speaking anxiety (Public Speaking Intervention Panel). We assessed the effects of the manipulated audience on behavior, physiological and subjective experiences. The corresponding panels are examples of variables in each cluster. See text and tables for full results of additional variables.

We first present the results from the within-subject comparisons of participants’ subjective experience (H1-H3; see Fig. 2; Table 1). As predicted by H1, participants perceived that the unsupportive audience required more cognitive, physical, and social energy than the supportive audience (cognitive: *F(1*,*76)* = 11.12, *p* = .001; physical: *F(1*,*76)* = 5.75, *p* = .019; social: *F(1*,*76)* = 5.28, *p* = .024). In addition, participants reported having experienced more communication anxiety symptoms when presenting in front of the unsupportive audience compared to the supportive audience. They felt the symptoms especially manifested in their language and behavior (*F(1*,*76)* = 6.90, *p* = .010) and thinking (*F(1*,*76)* = 16.83, *p* < .001), supporting H2a and H2b. Finally, participants reported greater levels of negative affect (*F(1*,*76)* = 8.09, *p* = .006) and emotional arousal (*F(1*,*76)* = 4.78, *p* = .032) after presenting to the unsupportive audience compared to the supportive audience, which aligned with H3a and H3b. H2c and H3c were not supported. However, the findings overall demonstrated that audience behavior affected peoples’ social-cognitive thought patterns, extending even to the level of perceptions of visceral and motor functions.

**Table 1:** Subjective experience by audience type (supportive vs. unsupportive).

Measure	Mean (Standard deviation)		F(1,76)	*p*
	Supportive audience	Unsupportive audience		
Thought-related symptoms	30.10 (10.80)	35.60 (12.80)	16.83	**< 0.001**
Cognitive demands	13.20 (5.92)	15.20 (6.11)	11.12	**0.001**
Subjective valence	2.12 (0.98)	2.58 (1.16)	8.09	**0.006**
Language-behavioral symptoms	25.30 (9.90)	27.70 (9.90)	6.90	**0.010**
Exertional demands	5.72 (3.21)	6.72 (3.51)	5.75	.**019**
Social demands	14.00 (4.01)	15.10 (4.06)	5.28	**0.024**
Subjective arousal	3.77 (1.09)	3.50 (1.05)	4.78	**0.032**
Physiological symptoms	2.68 (1.52)	2.86 (1.73)	0.85	0.361
Subjective dominance	3.27 (0.85)	3.18 (0.99)	0.60	0.442

Significant values are in [bold].

Note. Subjective Valence: 1 – Positive, 5 – Negative; Subjective Arousal: 1 – More Aroused, 5 – More Calm.

Next, we present audience effects on participants’ physiology and behavior (H4-H5, RQ1-RQ2; see Table 2). Speaking in front of the unsupportive audience elicited higher levels of arousal (*F(1*,*71)* = 12.05, *p* < .001) in the voice, supporting H4b. Interestingly, individuals expressed greater dominance in the voice (*F(1*,*71)* = 11.30, *p* = .001), and vocal valence did not differ by audience type while presenting in front of the unsupportive audience. Thus, H4a and H4c were not supported. Contrary to our predictions in H5, individuals’ breathing rate, heart rate, and pupil dilation did not vary by audience type. Regarding RQ1, we found that audience behavior significantly influenced individuals’ neural activity (measured via alpha-to-beta ratio; *F(1*,*72)* = 4.14, *p* = .046). For RQ2, speaking to the unsupportive audience decreased the speaking rate (*F(1*,*71)* = 8.11, *p* = .006). Other behavioral measurements did not differ by condition; however, participants who reported greater speaker anxiety prior to the intervention were less open and expressive nonverbally when presenting in front of the unsupportive vs. supportive audience (*r*_*expressiveness*_ = 0.28, *p*_*expressiveness*_ = 0.020; *r*_*openness*_ = 0.24, *p*_*openness*_ = 0.047). These results highlight how the (simulated) social audience behavior affects biological and behavioral and how a speaker’s temperament or personality shapes behavioral performance under stress.

We also assessed the effects of the VR-based public speaking tasks as a whole (RQ3-4). Completing the VR public speaking intervention lowered state anxiety overall (*M*_*post*_ = 33.4, *SD*_*post*_ = 9.34; *M*_*pre*_ = 38.1, *SD*_*pre*_ = 10.6; *t* = 5.67, *p* < .001), with effects more pronounced for those who reported higher levels of speaker anxiety prior to the virtual presentation (*F(1*,*220)* = 19.78, *p* < .001). In addition, participants were likely to recommend this VR-based public speaking simulation to others *(M* = 5.45 out of 7; *SD* = 0.64). Thus, this VR-based public speaking simulation shows promise as a platform for communication training, with the added benefit that it enables experiments on how social-cognitive processes influence physiology, and behavior.

**Table 2:** Differences in behavior & physiology by audience type (supportive vs. unsupportive).

Measure	Mean (Standard deviation)		df	F (*p*-value)
	Supportive audience	Unsupportive audience		
Voice: Arousal	0.34 (0.13)	0.36 (0.13)	(1, 71)	12.05 **(< 0.001)**
Voice: Dominance	0.38 (0.12)	0.40 (0.12)	(1, 71)	11.30 **(0.001)**
Speaking rate (wpm)	130 (23.30)	127 (24.60)	(1, 71)	8.11 **(0.006)**
Neural activity (alpha/beta)	6.07 (2.20)	7.32 (4.82)	(1, 72)	4.14 **(0.046)**
Voice: Valence	0.49 (0.10)	0.50 (0.09)	(1, 71)	2.46 (0.121)
Heart rate	93.40 (13.20)	94.70 (11.90)	(1, 52)	1.30 (0.260)
Gaze on audience vs. monitor	9.75 (24.30)	7.82 (9.50)	(1, 67)	0.50 (0.482)
Breathing per minute (bpm)	12.30 (3.83)	11.90 (3.67)	(1, 31)	0.36 (0.553)
Motion: Expressiveness	2.52 (1.46)	2.57 (1.52)	(1, 71)	0.34 (0.564)
Pupil dilation	3.73 (0.68)	3.76 (0.76)	(1, 62)	0.31 (0.579)
Gaze dispersion	35.60 (33.00)	34.70 (29.90)	(1, 74)	0.26 (0.610)
Motion: Openness	68.10 (13.10)	68.50 (13.40)	(1, 71)	0.15 (0.701)

Significant values are in [bold].

Note. df = degree of freedom; alpha/beta = electroencephalography (EEG) alpha-to-beta ratio; wpm = words per minute; bpm = breathing per minute.

## Discussion

Here we examined how participants responded to two social-communicative challenges in VR. We found that the virtual audience behavior strongly affected participants, demonstrating the value of VR as a social-cognitive experimentation tool, underscoring its value for training challenging public-speaking situations, and providing insights into the dynamics of speaker behavior during social-evaluative communication challenges. While the task was a scientific presentation, it serves as a robust model for a wide range of high-stakes speaking situations where audience feedback is critical. Our findings on this communication feedback loop between speakers and audiences are therefore relevant across a wide range of professional and academic contexts - from business pitches to job talks.

First, people’s subjective experiences generally supported our predictions. Participants reported exerting greater effort and experiencing more cognitive and behavioral anxiety symptoms when presenting in front of the supportive audience. They also felt more negative affect and emotional arousal for the unsupportive vs. supportive audience. These results demonstrate that the experimentally manipulated virtual audience had a strong influence on participants’ subjective experience and that - despite the virtual and ultimately artificial nature of this mediated communication challenge - it produced real-world effects^31^.

In addition, the physiological and behavioral measures offer insights into the speaker’s response to the virtual audience and show potential evidence of compensatory strategies. The combination of increased vocal arousal and dominance alongside a higher alpha-to-beta ratio might be interpreted as a compensatory vocal effort, perhaps as an attempt to re-engage the audience, coupled with cognitive disengagement due to withdrawing or re-allocating mental resources from the presentation content itself.

Though generally harder to interpret, the null findings are also informative. For instance, the lack of a significant difference in heart rate, despite the increase in vocal arousal, suggests a difference between the communicative compensation strategies and the systemic stress response. Similarly, the absence of an effect on gaze behavior may reflect the high cognitive load of the task: the need to manage slides and notes could have created a floor effect that might have masked subtler gaze aversion and apprehension patterns^9^. In sum, these results underscore that arousal and stress are not monolithic constructs, and that behaviors can vary by task demands. Importantly, we also found that individual differences moderated these behavioral responses; participants with higher levels of speaking anxiety became less open and expressive when facing the unsupportive audience, suggesting that pre-existing dispositions can amplify negative behavioral reactions to social threat.

By triangulating multiple measures – behavioral, physiological, and experiential – the current study provides a holistic picture of public speaking, one of the most important social communication skills across business, educational, and civic contexts. Methodologically, by simulating real-life communication environments and manipulating theoretical variables like audience supportiveness, we demonstrate the utility of VR-based simulations to studying complex social communication processes. This goes beyond existing work that has used VR to study people’s self-reported ratings of virtual audiences. Going forward, integrating VR-based training systems with real-time physiological and behavioral monitoring could lead to significant practical implications. For instance, future systems might monitor participants’ responses in real-time and provide adaptive feedback (e.g., virtual audience members smiling if the speaker is effectively using humor), personalized training (e.g., try “X” instead of “Y” here), or social support or specific symptom-provocations geared towards communication apprehension^25,32^.

However, as with all research, this study is not without limitations. For example, in the post-experimental interview, some speakers mentioned that despite the simulation being realistic and immersive, they were aware the audiences were not real. Some speakers also mentioned that it would have been helpful if the audience behavior changed based on their performance as we suggested above future systems might.

Another limitation stems from our within-subject design, in which presentations were delivered successively. Although participants removed the headset to fill out survey questions, this raises the risk that the emotional state induced by the first condition could have influenced the speaker’s response to the second. While our counterbalancing and statistical controls for presentation order mitigate this risk, future studies aiming to validate VR platforms specifically as training tools should consider between-subjects designs to fully isolate the effects of audience condition.

Another methodological consideration relates to our sampling approach: Our study did not employ clinical exclusion criteria. This was a deliberate choice aimed at capturing the full spectrum of public speaking response in our university-affiliated sample – our target for generalizability of the science communication task. However, we acknowledge that this means our results describe dynamics of social-evaluative threat in a non-clinical sample. While the demanding nature of the task likely deterred individuals with debilitating anxiety from participating, the findings should not be generalized to clinical populations.

Lastly, most of the physiological and behavioral measures were aggregated across participants. Future work could examine individualized responding and trace dynamic changes over the duration of the presentation.

Going forward, given the rapid developments towards high-realism avatars and LLM-based agents, often combined with VR^33,34^, we see a lot of potential to create more flexible and realistic audiences. For example, it would be important and feasible, to not only simulate the public speech itself, but also the Q&A session afterwards, as this session is a more bi-directional communication situation and one in which speakers are on the spot and may receive completely unanticipated questions.

Furthermore, while our multi-modal measurement approach is currently still resource-intensive, we believe it can serve as a blueprint for future communication training technologies. Specifically, the rapid integration of biosensors, eye-tracking, and body-tracking into consumer VR/AR headsets will soon make a comprehensive, real-time data collection much easier and likely commonplace. With this in mind, our work demonstrates how integrated physiological and behavioral data can be used to understand a speaker’s state, paving the way for adaptive, next-generation training and feedback systems.

## Conclusion

The current study examined how audience behavior in immersive VR impacts the speaker. The findings generally supported our predictions about audience effects on subjective experiences and shed insights into the speakers’ physiological and behavioral responses. The results, including the null findings (e.g., no significant differences in the heart rate or gaze), underscored the complexity of the public speaking process and that physiological and behavioral responses can vary by task demands. In addition to demonstrating how we can use immersive VR as an experimental tool to decipher social-cognitive mechanisms, this study also highlights how VR-based experimental paradigms can be integrated with subjective, physiological, and behavioral measures to create synergy between stimulus delivery, online social cognition, and communication outcomes, and between experimental control and ecological validity. Finally, understanding social-cognitive mechanisms of public speaking holds immense practical significance. Public speaking anxiety affects many individuals and public speaking skills are a top-ranked 21st-century job skill. Therefore, leveraging VR to enhance communication skills in speakers – through training, feedback, and intervention – is a burgeoning area where insights from communication science and biology can yield tangible benefits.

## Methods

The study was approved by the Institutional Review Board at Michigan State University [STUDY00007966] and conducted in accordance with relevant guidelines and regulations. We obtained informed consent and collected data from 80 participants (*M*_*age*_: 32.28, *SD*_*age*_: 10.60, range_age_: 20–75; 33 Male, 43 Female, 4 Nonbinary). We recruited participants who have presented or will need to present science or research-based work, which included graduate students, postdoctoral researchers, and faculty. All participants received monetary reimbursement for their participation. Sample size was determined a-priori based on a power analysis for a paired-sample *t*-test (*α* = 0.05, *1-β* = 0.8). We powered the study to detect a small-to-medium-sized effect (*d* = 0.3), which was chosen as a conservative estimate and as the smallest effect of practical interest; while literature on in-person social-evaluative stressors often reports larger effects, we anticipated that responses might be attenuated in VR. This analysis suggested a sample size of 71 participants.

### Software and equipment

**Fig. 3:**
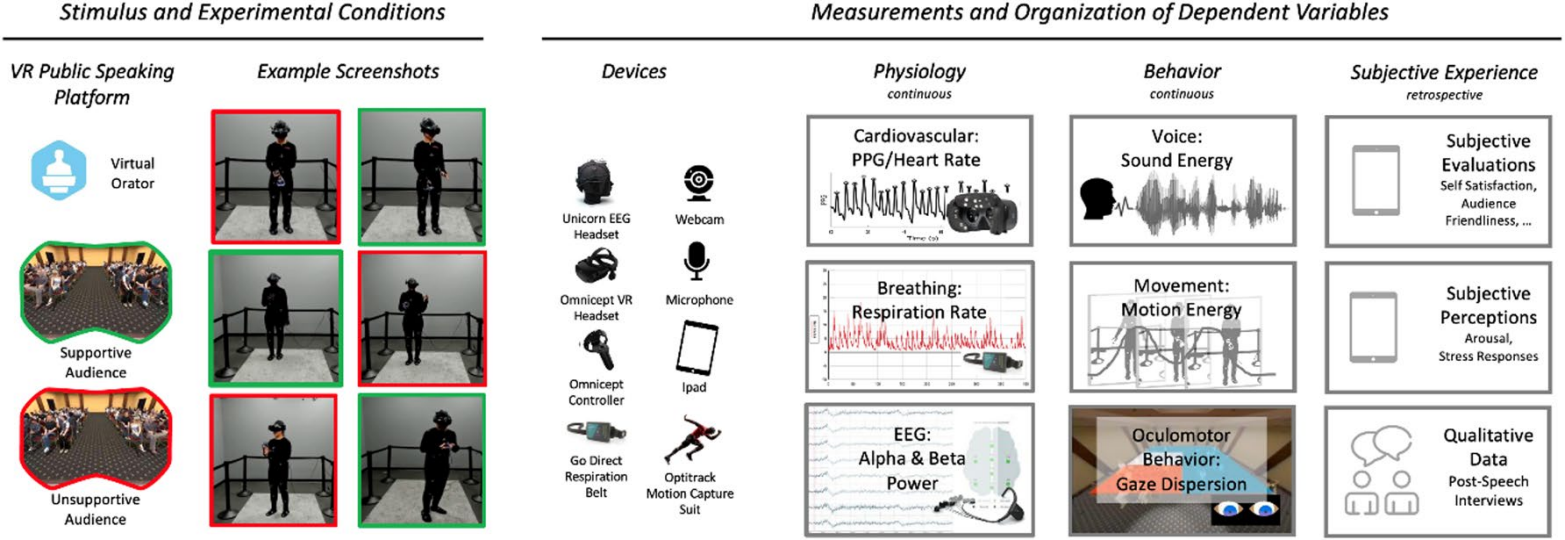
Overview of mthodology. Participants gave two scientific talks while wearing the HP Omnicept VR headset. Their physiology and behavior were recorded via the respective measurement gear, and they reported their subjective experiences through the post-presentation questionnaires. The independent variable was the type of audience (manipulated in the Virtual-Orator platform), and the dependent variables were the physiological, behavioral, and subjective responses measured, enabling a comprehensive assessment of the complex public speaking processes.

See Fig. 3 for an overview of the software and measurement equipment used. We used the Virtual-Orator public speaking software^35^ as the VR-based experimental platform. This software allowed us to create the computer-generated audiences and extract general gaze behavior while completing the public speaking tasks. For the head mounted display, we used the HP Reverb G2 Omnicept. The headset included pupil dilation and heart rate measurement capabilities. In addition, we used the GTec Unicorn mobile cap to collect electroencephalography (EEG) data and Vernier Respiration Belt to measure the breathing rate. Furthermore, the Optitrack motion capture suit recorded participants’ nonverbal behavior.

### Experimental conditions

We used Virtual Orator software to generate the supportive and unsupportive audiences. Specifically, we adjusted the audience parameters, ‘audience friendliness’, ‘audience interest’, ‘audience concentration’, and ‘general distraction’ for each type of audience. For the supportive audience, these parameters were all set to the maximum levels, resulting in audience behaviors that signaled attentiveness and friendliness to the speaker. These settings generated the attentive audience, who looked at the speaker or the projector with the slides, had an upright body posture, and neutral and friendly expressions, and did not make any distracting noise. The unsupportive audience, on the other hand, was generated by setting the respective software controls to the lowest levels, resulting in an audience that appeared distracted and unfriendly. In this condition, audience members turned away from the speaker (as if talking to the person behind them), frequently checked their cell phones, slouched, and fell asleep. Beyond audience behavior, the unsupportive condition also included loud environmental noise upon entering the room and cell phone alert and talking sounds during the presentation.

As manipulation check, we asked participants a series of audience evaluation questions in the post-presentation survey after each presentation. Specifically, participants were asked to rate the audience on seven adjectives (e.g., attentive, friendly, cold, ranging from 1 - not at all to 7 - very much). The paired *t*-tests showed that participants perceived the supportive and unsupportive audiences accordingly (see Table 3), demonstrating that our manipulations worked as intended.

**Table 3:** Results from manipulation check (paired *t*-test).

Measure	Mean (Standard deviation)		Effect Size		t (*p*-value)
	Supportive audience	Unsupportive audience	d	95% CI	
Audience: Attentive	4.37 (1.86)	2.65 (1.65)	0.78	[0.53, 1.03]	6.90 (**< 0.001**)
Audience: Polite	4.74 (1.40)	2.91 (1.50)	0.93	[0.67, 1.20]	8.25 (**< 0.001**)
Audience: Rude	2.08 (1.28)	3.90 (1.90)	0.87	[0.61, 1.13]	-7.68 (**< 0.001**)
Audience: Interested	4.28 (1.76)	2.77 (1.47)	0.72	[0.47, 0.97]	6.34 (**< 0.001**)
Audience: Friendly	4.13 (1.75)	2.92 (1.57)	0.62	[0.38, 0.86]	5.48 (**< 0.001**)
Audience: Cold	3.56 (1.71)	4.76 (1.69)	0.49	[0.26, 0.73]	-4.36 (< **0.001**)
Audience: Lively	3.46 (1.73)	2.77 (1.79)	0.36	[0.13, 0.59]	3.20 (**0.002**)

Significant values are in [bold].

Note. d = Cohen’s d; CI = Confidence Interval.

### Procedures

This study employed a within-subject design in which all participants gave two presentations, one to a supportive vs. unsupportive audience. This design was chosen for its statistical power to control for the large individual differences inherent in public speaking competency and its physiological correlates. By having each participant act as their own control, we could more sensitively isolate the effects of the audience manipulation. The order of these presentations was counterbalanced across participants to mitigate potential carry-over effects (e.g., habituation or contrast effects) so that half of the sample started with an unsupportive audience, the other half with the supportive audience.

Participants filled out a pre-survey and were instructed to prepare two different 8–12 min scientific presentations before coming into the lab. They choose topics with which they were familiar, particularly their own research. This also ensured that the presentation content was novel and different for each condition. After participants arrived at the lab, provided consent to the study, and watched a brief explanation of the study via a video clip, we equipped the participants with the ambulatory measurements (motion capture suit, EEG, breathing belt) and the HP Omnicept VR headset and calibrated the eye-tracking system within the headset. Then, participants entered the Large-Hotel-Meetingroom-With-Chairs-environment of the Virtual-Orator public speaking platform^35^. For each 8-12-minute presentation, we loaded the participants’ slides into the Virtual-Orator system; these slides were displayed on a virtual laptop in front of them and on the two projectors on their left and right sides. Once participants finished their presentation, they completed a post-presentation interview and survey.

### Main measures

#### Subjective measures and anxiety instruments

We included three subjective measures. First, we adopted and modified the cognitive, exertional, and social subdimensions of a video game demand scale^36^, which asked participants to rate items on a 7-point Likert scale (1 - strongly disagree to 7 - strongly agree). The cognitive demand subscale included four statements about how much thinking was required by the public speaking task (e.g., “*Presenting in VR was cognitively demanding*”). The exertional subdimension comprised the following two items related to physical energy required by the tasks (e.g., “*I felt physically exhausted after delivering the presentation*”). The social demand subscale included three items about how much the public speaking task made the participants react to the audiences (e.g., “*Being with the audience in the room had an impact on how I gave the presentation*”).

To measure individuals’ subjective affective responses, we adopted the self-assessment manikin (SAM^37^. Three SAM categories asked participants to select the picture of the manikin that best represented their feelings of valence (1 - most positive to 5 - most negative), arousal (1 - most aroused to 5 - least aroused), and dominance (1 - least dominant to 5 - most dominant) during the presentation. These three items represented the Subjective Valence, Subjective Arousal, and Subjective Dominance measures.

For the anxiety-related symptoms, we asked participants to report to what extent they experienced language-behavioral, thought-related, and physiological symptoms while presenting, on a scale from 1 (not at all) to 7 (a lot). The language-behavioral subdimension included nine symptoms such as “*I misspoke or stuttered*” and “*I made displacement activities (scratching head*,* touching face*,* tapping*,* etc.).*” Thought-related symptoms comprised 10 items including “*Unwanted thoughts came to my mind during the presentation*” and “*I made negative evaluations of myself.*” Perception of physiological alterations was measured by one item, “*I experienced physiological symptoms or thoughts that interfered with my performance.*”

In addition to the subjective measures, we included two additional scales about state anxiety and speaker confidence levels. To measure individuals’ state anxiety levels before and after the VR-based intervention, we used 20 items from the State Trait Anxiety Inventory^38^. These statements asked participants to indicate how much they feel specific emotions from 1 (Not at all) to 4 (Very much so). For baseline speaker confidence level, we adopted the 12-item Personal Report of Confidence as a Speaker (PRCS) scale^39^, which asked participants to rate each statement from a scale of 1 (not at all characteristic of me) to 5 (extremely characteristic of me).

#### Physiological and behavioral measures

Over-time measures of participants’ neurophysiological and visceromotor responses included heart rate and pupil dilation (measured via the HP Omnicept Recorder), EEG (measured via a GTec Unicorn mobile EEG system), and breathing rate (measured via a Vernier respiration belt). Measures of participants’ behavior as they deliver the speech and respond to the audience included motion (measured via the motion-capture system), gaze behavior (generated by the Virtual-Orator platform), and their speech patterns (analyzed from presentation recordings).

### Data processing and analysis

The processed data and scripts are available online (github.com/nomcomm/speaker_responses_ audience). An overview of the main derived metrics for biological and behavioral data is shown in Fig. 2. In brief, all data streams were related to the common onset point - the moment participants entered the conference room. They then immediately started their speech, and the moment was marked in all separate data streams. We then extracted relevant metrics and averaged them over the first 3 min of each speech (i.e., one average heart rate for the presentation in front of a supportive audience and one average HR for the presentation in front of an unsupportive audience). The only exceptions to the 3-minute aggregate were gaze dispersion and screen-to-audience gaze ratio measures. These metrics were computed for the entire duration by the Virtual-Orator system. We decided on the 3-minute cut-off after examining the metrics over-time in 30 s increments. We noticed that by the 180 s mark, the metrics were generally consistent, excluding certain moments of peaks and valleys. We will analyze these patterns in more detail in a follow-up paper.

Heart rate and pupil dilation metrics were directly computed by the HP Omnicept recorder software. EEG data were processed in MATLAB, using the unicorn python package to read in the data, and then using EEGLAB’s filtering, automated artifact correction, and power spectrum algorithms to compute global alpha and beta power spectrum values for each speech^40^. Then, for each data point, the alpha-to-beta ratio was calculated (alpha/beta). We note that while the alpha/beta ratio provides only a coarse and indirect measure of relaxed vs. active cortical states and should be interpreted with caution^41,42^, the design of our study makes it feasible to track changes within participants (i.e., supportive vs. unsupportive audience).

Breathing rate was determined via the Vernier Graphical breathing belt analysis kit. Eye gaze was quantified via Virtual Orator’s integrated audience-zone analysis tool, which measured time spent looking at each of six audience sections (front left, front middle, front right, back left etc.). We computed the standard deviation of those metrics as a measure of equality of gaze allocation to all zones, which contained the same number of audience members. Furthermore, we used the motion-capture system to track people’s nonverbal movements throughout the speech and used python code to calculate expressiveness and openness indices. Lastly, we analyzed valence, arousal, and dominance in people’s voices using the wav2vec model^43^.

For statistical analysis of the main measures, we fitted a linear mixed effects model for each metric, with audience type as the main effect variable. We note that our hypotheses and analysis plan were specified a priori, though not formally pre-registered. Intercepts varied by participant to consider the repeated-measure design, and we controlled for potential confounding effect of the speech order (i.e., which audience participants encountered first). In the spirit of transparency and reproducibility, anonymized data and analysis scripts are available online (see Data and Code Availability). We cleaned the data before fitting the models based on the following procedure: For each metric, if the data for one speech was missing, we excluded the participant all together. This ensured adequate within-subject comparisons.

## Data Availability

The anonymized dataset and data analysis scripts are available via GitHub at github.com/nomcomm/speaker_responses_audience.
